# Effect of khat *(Catha edulis)* chewing on liver function markers among healthy males: a comparative cross-sectional study

**DOI:** 10.11604/pamj.2026.53.27.49553

**Published:** 2026-01-21

**Authors:** Ahmed Ismail Mohamed, Jama Mohamed, Mustafe Mohamed Hussien, Hussein Mohamoud Nour, Abdulahi Abdiwali Mahamed, Ahmed Ibrahim Farah

**Affiliations:** 1 Faculty of Human Nutrition and Dietetics, College of Medicine and Health Science, University of Hargeisa, Hargeisa, Somaliland,; 2 Faculty of Medical Laboratory Science, College of Medicine and Health Science, University of Hargeisa, Hargeisa, Somaliland,; 3 School of Medical Laboratory Science, Edna Adan University, Hargeisa, Somaliland,; 4 Faculty of Data Science and Statistics, College of Business, Economy, and Statistics, University of Hargeisa, Hargeisa, Somaliland,; 5 Department of Nutrition, College of Health Science, Admas University, Garowe, Somalia

**Keywords:** Khat chewing, biochemical markers, liver function markers

## Abstract

**Introduction:**

khat (Catha edulis) chewing is a prevalent practice in Somaliland, yet its impact on liver function among metabolically healthy males remains inadequately understood. This study aims to evaluate the effects of chronic khat chewing on liver function markers in the Somaliland population.

**Methods:**

a comparative cross-sectional study was conducted in Somaliland from January to March, 2024. A sample of apparently healthy males, consisting of habitual khat chewers and non-chewers, was selected using purposive sampling and convenience sampling techniques, respectively. Liver function markers analyzed included total bilirubin, total protein, albumin, and glutamate pyruvate transaminase (GPT). Data were analyzed using descriptive statistics and inferential tests to compare the two groups, with significance set at p < 0.05.

**Results:**

chronic khat chewers exhibited higher total bilirubin levels (p = <0.001) and lower total protein levels (p = <0.001) compared to non-chewers, suggesting potential hepatotoxic effects. No significant differences were observed in the serum levels of albumin and GPT between the two groups.

**Conclusion:**

chronic khat chewing is associated with significant alterations in liver function markers, particularly elevated total bilirubin and reduced total protein levels, indicating potential hepatotoxic effects. However, albumin and GPT levels remained unaffected. Public health authorities should implement educational campaigns to raise awareness about the potential adverse effects of Khat on liver health. Healthcare providers should consider monitoring liver function markers in habitual Khat chewers as part of routine health assessments.

## Introduction

*Catha edulis* is an evergreen shrub or small to medium-sized tree that is a member of the Celastraceae family. It is sometimes referred to as Khat, qat, or Khat [1]. It is mostly grown as a tiny tree or bush in Yemen and other East African nations [2,3]. Previously, the Khat tree originated from Ethiopia, more precisely in Hararghe, and gradually spread to Yemen, Somalia, Sudan, South Africa, Madagascar, Afghanistan, and Turkestan [4].

Cathinone, the primary active ingredient obtained from chewing Khat, causes euphoria, alertness, and stimulation of the central nervous system, which makes this practice popular with a significant percentage of society. People also think that because khat gives them more energy and alertness, it makes them more productive, especially when working by hand. Chewing Khat, however, has been linked to negative impacts on normal physiology [5]. In Somaliland, chewing Khat has been a serious national issue for the last fifty years. Many individuals, especially medical professionals, think that chewing khat might harm one's health and have serious social, financial, and medical repercussions [6]. While khat chewing is not popular throughout the world, published literature is scarce on the medical consequences of khat consumption. However, there are some studies that document a link between khat chewing and the incidence of myocardial infarction, dilated cardiomyopathy, vascular disease such as hypertension, cerebrovascular ischemia and thromboembolism, diabetes, sexual dysfunction, duodenal ulcer, and hepatitis [7,8].

The liver is particularly susceptible to the negative consequences of khat consumption [9]. Several metabolic disturbances of the liver resulting from Khat leaves have been documented in experimental studies in animals [10]. Several studies have concluded that Khat consumption causes subclinical and clinical liver damage; however, they all emphasize hepatocellular enzymes such as ALT, AST, GGT, and ALP [9,11-13]. To the best of our knowledge, no study has yet shed light on the effect of khat chewing on the synthesis and excretory activity of the liver. Therefore, this study aimed to assess the effect of khat chewing on albumin levels, total protein, total bilirubin, and glutamate oxaloacetate transaminase (GPT).

## Methods

**Study design and setting:** from January to March 2024, a comparative cross-sectional study was conducted. Information on the research settings was published elsewhere [14]. This study was briefly carried out in Southern District in Hargeisa, the capital city of Somaliland.

**Study subjects:** this study enrolled apparently healthy adult males aged 18 years and above. The Khat chewer (exposed) group was recruited using a purposive sampling technique to specifically identify individuals with a well-defined history of chronic Khat use (see inclusion criteria below). The non-chewer (non-exposed) group was recruited using a convenience sampling technique from the same community settings to ensure geographical and socio-demographic comparability. This mixed-method approach was employed due to the high prevalence of khat chewing in the study area, making it challenging to find a sufficient number of eligible non-chewers through random or purely purposive methods. Efforts were made to match the groups broadly on age and area of residence to enhance comparability. Subjects who agreed to participate in the study were contacted by the authors and asked to come to the laboratory department at Edna Adan University to sign consent forms, begin data collection, and provide blood samples.

**Sample size determination:** the sample size for this comparative cross-sectional study, assessing the effect of chronic Khat chewing on liver function markers among apparently healthy males, was determined using a significance level (α) of 0.05, a power (1 - β) of 80%, a moderate effect size (Cohen's d) of 0.5, and a standard deviation of 1. A one-tailed two-sample independent t-test was employed to examine the hypothesis that Khat chewing increases liver function markers. The formula for calculating the sample size in each group was as follows:


$$n=\frac{2{\left(z_{\alpha }+z_{\beta }\right)}^{2}\sigma^{2}}{d^{2}}$$

Where n is the required sample size per group. Z_α_ is the critical Z-score for the chosen alpha (0.05). Z_β_ is the critical Z-score for the desired power (0.80). σ represents the standard deviation (assumed to be 1 in this case). d is the effect size (0.5 in this study). Substituting the values, we calculated that the required sample size for each group was approximately 51. Since there are both an exposed group and a non-exposed group, the total sample size amounts to 102, with 51 participants in each group. This sample size is expected to provide adequate statistical power to detect the anticipated effect while maintaining a significance level of 0.05.

**Participant selection and definition of exposure:** inclusion criteria for Khat chewers (exposed group). To be included in the exposed group, participants had to be apparently healthy adult males who met the following operational definition of a “chronic Khat chewer”: i) frequency: chewing Khat at least two days per week; ii) duration: a history of regular khat chewing for a minimum of two consecutive years; iii) intensity: consuming more than 250 grams of fresh Khat leaves per typical chewing session.

**Inclusion criteria for non-chewers (non-exposed group):** participants in the non-exposed group were apparently healthy adult males who reported never having chewed Khat in their lifetime.

**Exclusion criteria:** both groups were designed to minimize the influence of common non-Khat-related factors on liver function. Exclusion criteria included: a history of known liver disease (e.g., cirrhosis, fatty liver disease) or hepatitis; current use of hepatotoxic medications (e.g., paracetamol/acetaminophen, anti-tuberculosis drugs, certain antibiotics); diagnosis of any chronic metabolic disorder (e.g., diabetes, hypertension); self-reported alcohol consumption; and a body mass index (BMI) ≥ 30 kg/m^2^ to exclude individuals with potential non-alcoholic fatty liver disease (NAFLD).

**Assessment of outcomes:** the outcome variables of the study were liver function markers, including serum levels of glutamate pyruvate transaminase (SGPT), albumin level in the blood, total protein level, and serum levels of total bilirubin.

**Blood sampling:** venipuncture was done in the late evening. Five milliliters of venous blood were drawn from each subject. Blood samples were labelled and coded in the same way that each subject's data form was. After centrifugation at 2500 rpm for 10 minutes, serum was isolated from the whole blood. The samples were kept in the refrigerator at +4C0 until they were analyzed. The biochemical markers of liver function (GPT, bilirubin, total protein, and albumin level) were measured using a clinical chemistry analyzer (BS-240 Mindray).

**Reagents and procedures:** total serum protein, total albumin level, total bilirubin level, and glutamate pyruvate transaminase (GPT) were examined by enzymatic methods using diagnostic kits obtained from Excel “Diagnostic”, Italia. Reagents were freshly prepared, and the procedures were performed in accordance with standard operating procedures. Each sample was analyzed two times to ensure the consistency of the test.

**Statistical analyses:** SPSS software, version 27, was used for the statistical analyses. Continuous data were summarized as mean ± standard deviation (SD) for normally distributed variables and as median ± interquartile range (IQR) for non-normally distributed variables. Categorical variables were represented by frequency and percentages. Normality of continuous variables was assessed using the Kolmogorov-Smirnov and Shapiro-Wilk tests. For variables that followed a normal distribution, comparisons between Khat chewers and non-chewers were performed using the independent samples t-test. For non-normally distributed variables, the Mann-Whitney U-test was applied. Statistical significance was set at p ≤ 0.05.

**Assessment of confounding factors:** to enhance the internal validity of the study and strengthen the association between khat chewing and liver function markers, we employed both design and statistical strategies to account for potential confounding factors. During participant recruitment, we used a detailed questionnaire to collect data on factors known to affect liver function, including medical history, current medication use, alcohol consumption, and smoking status. Individuals with conditions or behaviors like known liver disease, hepatotoxic medication use, alcohol consumption, and obesity (BMI ≥ 30) were excluded. Furthermore, we collected anthropometric measurements (height and weight) to calculate BMI for all participants to enforce the obesity exclusion criterion. While the primary analysis focused on group differences (chewers vs. non-chewers), the sociodemographic matching during recruitment and the exclusion of major confounders increase the likelihood that the observed differences in bilirubin and total protein are attributable to Khat chewing.

**Ethical approval:** before beginning the study, the Research Ethics Review Committee of the University of Hargeisa provided ethical approval (ethical approval No. DRCS/51/01/2024). Furthermore, each study participant obtained clear verbal informed consent before participation. To preserve the participants' privacy, the confidentiality of the information acquired from them was strictly maintained throughout the study. Furthermore, participants were told of their individual results at the start of the study and were given a summary of the findings at the end, assuring transparency and adherence to ethical guidelines.

## Results

**Sociodemographic characteristics of participants (Khat chewers and non-chewers):** a total of 104 subjects, 52 Khat chewers (exposed group) and 52 non-chewers (non-exposed group), were enrolled in the study. The mean age for both groups was 42.13 years (SD ± 11.89). A total of 42 (40.4%) of the subjects were older than forty-four years, while only five subjects (4.8%) were aged between 18 and 24 years. Regarding their educational status, 27 subjects didn´t receive any formal education, while 24% had a tertiary level of education. In terms of marital status, the majority of subjects, 73 (70.2%), were married with children. Furthermore, the majority, 40 (38.5%), of the study participants were self-employed, whereas only nine subjects were government employees. The details are shown in Table 1.

**Table 1:** sociodemographic characteristics of participants (khat chewers and non-chewers)

Variable	Categories	Frequency	Percentage
**Age (years)**	18-24	5	4.8%
	25-34	29	27.9%
	35-44	28	26.9%
	45+	42	40.4%
**Level of education**	No formal education	27	26%
	Primary education	27	26%
	Secondary education	25	24%
	Tertiary education	25	24%
**Marital status**	Married with children	73	70.2%
	Married without children	6	5.8%
	Single	25	24.0%
**Employment**	Government employer	9	8.7%
	Private employer	38	36.5%
	Merchant	11	10.6%
	Self-employed	40	38.5%
	Unemployed	6	5.8%

**Chewing characteristics and lifestyle behaviors among Khat chewers:** the average age at which individuals start chewing Khat is 26.54 years, with a standard deviation of 8.9 years. The vast majority of Khat chewers (82.7%) engage in this behavior daily. Most individuals (92.3%) spend over 3 hours on a single chewing session. When it comes to the amount consumed per session, 44.2% consume less than 250 g of Khat, while 34.6% consume between 250 g and 500 g. The reasons for Khat chewing vary among individuals. Some chew Khat to increase energy (21.2%), feel refreshed (28.8%), increase alertness (5.8%), or stay awake (5.8%). A moderate percentage (34.6%) report addiction as a reason, while only 3 (5.8%) chew for book reading.

The majority of chewers (90.4%) prefer jebis khat, while a smaller percentage (9.6%) opt for other types. Notably, some individuals combine Khat chewing with other substances. Specifically, 34.6% consume cigarettes, 23.1% consume energy drinks, and additional substances such as tea and coffee are also consumed. Regarding sleep patterns, 71.2% of Khat chewers sleep less than 6 hours during the night. Among them, 23.1% sleep between 6 and 8 hours, while 5.8% sleep more than 8 hours. During the day, 50.0% of Khat chewers do not engage in daytime sleep, with 11.5% reporting sleep between 1 and 3 hours, and 38.5% sleeping more than 3 hours (Table 2).

**Table 2:** chewing characteristics and lifestyle behaviors among khat chewers

Variable	Categories	n (%)
**Age at start of khat, Mean (SD)**		26.54 ± 8.90
**Chewing frequency**	Once a week	3 (5.8%)
	Two times per week	3 (5.8%)
	> Three times per week	3 (5.8%)
	Daily	43 (82.7%)
**Time spent on single chewing**	≤ 3 hours	4 (7.7%)
	> 3 hours	48 (92.3%)
**Amount of khat chewed per single consumption**	< 250 g	23 (44.2%)
	250 g - 500 g	18 (34.6%)
	> 500 g	11 (21.2%)
**Reason for khat chewing**	Increase energy	11 (21.2%)
	Makes me feel refreshed	15 (28.8%)
	Increases alertness	2 (3.8%)
	Stay awake	3 (5.8%)
	Addiction	18 (34.6%)
	Reading books	3 (5.8%)
**Type of khat**	Jebis	47 (90.4%)
	Others	5 (9.6%)
**Other material on khat chewing**	Cigarette	18 (34.6%)
	Energy drinks	22 (42.3%)
	Others (tea, coffee, etc.)	12 (23.1%)
**Night sleep hours**	< 6 hours	37 (71.2%)
	6 - 8 hours	12 (23.1%)
	> 8 hours	3 (5.8%)
**Day sleep hours**	None	26 (50.0%)
	1-3 hours	6 (11.5%)
	> 3 hours	20 (38.5%)

**Biochemical markers of the liver among Khat chewers and non-chewers:** the Kolmogorov-Smirnov test and Shapiro-Wilk test were conducted to assess the normality of the data distribution for each biochemical marker in both the chewers and non-chewers groups (Table 3). The statistical tests indicate that the data distributions for total bilirubin, total protein, and GPT deviate from normality in both the chewers and non-chewers groups. However, the data distribution for albumin shows no significant departure from normality in both groups. These findings provide insights into the normality assumptions for further statistical analyses involving these variables.

**Table 3:** normality tests in biochemical tests

	Group	Kolmogorov-Smirnov			Shapiro-Wilk		
		Statistic	df	Sig.	Statistic	df	Sig.
Total bilirubin	Chewers	0.153	52	0.004	0.949	52	0.027
	Non-chewers	0.231	52	0.000	0.808	52	0.000
Albumin	Chewers	0.083	52	0.200	0.973	52	0.273
	Non-chewers	0.107	52	0.200	0.962	52	0.092
Total protein	Chewers	0.107	52	0.194	0.961	52	0.088
	Non-chewers	0.120	52	0.060	0.944	52	0.016
Glutamate pyruvate transaminase	Chewers	0.221	52	0.000	0.858	52	0.000
	Non-chewers	0.222	52	0.000	0.872	52	0.000

**Figure 1:**
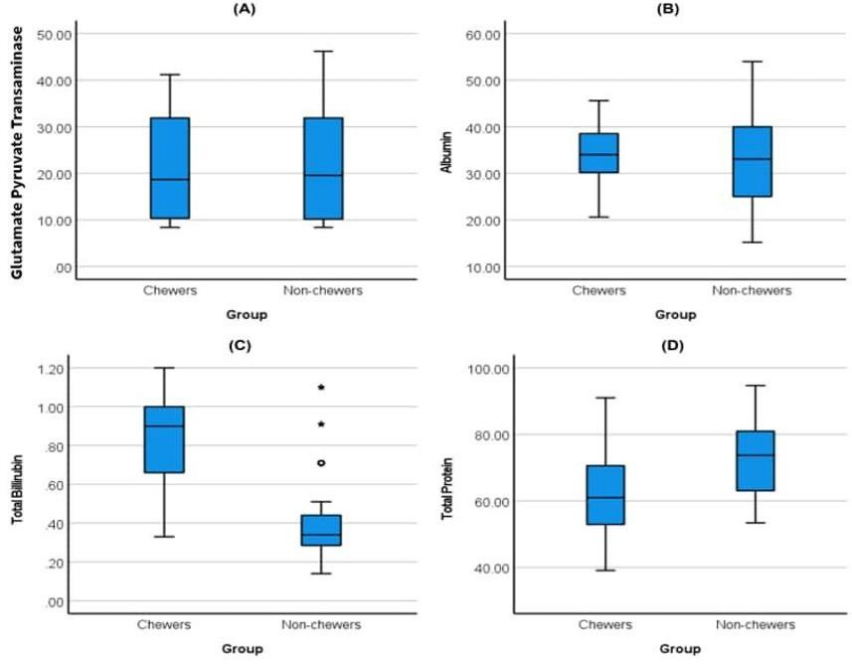
distribution of biochemical tests among khat chewers and non-chewers

Figure 1 shows a series of box plots comparing four biochemical parameters between chewers and non-chewers. For GPT (A), both groups have similar distributions of GPT levels, with comparable median levels and spread (interquartile range), indicating no significant difference between the two groups. For albumin (B), the median albumin levels are slightly higher in chewers compared to non-chewers, and non-chewers show a wider range, suggesting greater variability in this group. In the case of total bilirubin (C), there is a noticeable difference, with chewers having higher median total bilirubin levels and a broader distribution, while non-chewers have lower and less variable levels, along with some outliers indicated by asterisks and circles. For total protein (D), non-chewers have a higher median, and the range of total protein levels is similar for both groups, suggesting a substantial difference.

To further examine these differences, we reported the results from two-sample independent t tests for albumin and total protein and Mann-Whitney U-tests for total bilirubin and GPT in Table 4. The results confirmed that there is no significant difference (P = 0.421) in albumin level between Khat chewers and non-chewers. In contrast, the study shows that Khat chewers have significantly lower total protein levels compared to non-chewers (P = <0.001). In addition, Khat chewers have significantly higher total bilirubin levels compared to non-chewers (P = <0.001). Finally, the GPT level shows an insignificant difference between the two groups (P = 0.966).

**Table 4:** biochemical tests in khat chewers and non-chewers

	khat chewers	Non-chewers	P-value
Albumin (g/dl)	34.06 ± 6.25	32.83 ± 9.07	0.421
Total bilirubin (mg/dl)	0.84 ± 0.23	0.40 ± 0.22	<0.001*
Total protein (g/dl)	61.29 ± 13.32	73.59 ± 12.48	<0.001*
GPT (U/l)	21.09 ± 11.62	21.13 ± 11.62	0.966

* Significant difference between means using the independent samples test

## Discussion

The current study aimed to determine whether long-term chewing of Khat harms the production and excretory activity of the liver or not. The findings of this study revealed that Khat chewing significantly increases the total bilirubin level of the blood compared to the non-exposed group. The mean difference of bilirubin levels among chewers and non-chewers was 0.84 ± 0.23 mg/dl and 0.40 ± 0.22 mg/dl, respectively. However, other studies [15] documented that Khat chewing does not affect direct and indirect bilirubin levels of the blood. This could be explained by the fact that the toxic components in Khat leaves can temporarily or permanently stress the hepatocytes, leading to abnormal metabolism of bilirubin.

The study also found that the exposed group (Khat chewers) and the non-exposed group (Khat non-chewers) had significantly different mean levels of total serum protein. According to the study, Khat chewing dramatically lowers total protein levels. Nevertheless, there are contradictory studies or no studies to support this claim. This may be explained by the possibility that the chemical makeup of Khat leaves reduces the hepatocytes' capacity for synthesis. Furthermore, the research indicated that there is no significant mean difference in serum level of glutamate pyruvate transaminase (sGPT) and Albumin level. Meanwhile, other studies concluded that Khat chewing causes hepatocellular damage by increasing the levels of serum transaminases [9,11].

While these findings contribute to the growing body of literature on the health effects of Khat chewing, several limitations should be acknowledged. The study's small sample size and observational design may limit the generalizability of the results. Furthermore, this study focused on GPT (ALT) as a marker of hepatocellular injury. The inclusion of other standard liver enzymes, such as aspartate aminotransferase (AST) and alkaline phosphatase (ALP), would have provided a more comprehensive profile of liver damage, potentially differentiating between hepatocellular and cholestatic injury. Future studies should include a broader panel of liver enzymes to fully characterize the hepatotoxic profile of Khat.

Moreover, the use of non-probability sampling methods (purposive for chewers and convenience for non-chewers) introduces a potential for selection bias. Participants were not randomly selected from the general population, which may limit how representative our sample is of all Khat users and non-users in Somaliland. For instance, the purposive sampling of chronic chewers might have over-represented individuals with more severe habits or those more readily accessible to researchers. Similarly, the convenience sample of non-chewers may not fully represent the broader non-chewing population. Additionally, the resource limitation prevents a comprehensive implication of Khat chewing. Future research with larger sample sizes and more diverse populations, as well as longer study durations, should include a broader panel of liver enzymes to fully characterize the hepatotoxic profile of Khat and validate and expand upon these findings.

## Conclusion

In this comparative cross-sectional study, we evaluated the impact of Khat (*Catha edulis*) chewing on liver function markers among metabolically healthy males. Our findings indicate that chronic khat chewing is associated with significant alterations in certain liver function markers. Specifically, khat chewers exhibited higher total bilirubin levels and lower total protein levels compared to non-chewers, suggesting potential hepatotoxic effects of khat. However, no significant differences were observed in the serum levels of albumin and GPT between the two groups. Given these findings, it is recommended that public health authorities in regions where khat chewing is prevalent implement educational campaigns to raise awareness about its potential adverse effects on liver health. Additionally, healthcare providers should consider monitoring liver function markers in habitual Khat chewers as part of routine health assessments. Further research with larger and more diverse populations is essential to confirm these results and elucidate the underlying mechanisms of khat-induced liver damage. Longitudinal studies could provide more comprehensive insights into the long-term effects of khat chewing on liver function and overall health.

### What is known about this topic

- Chewing khat (Catha edulis) is a prevalent practice in East African and Arabian countries, known for its stimulant effects due to the active ingredient cathinone;
- Khat consumption has been linked to various adverse health effects, including cardiovascular, metabolic, and gastrointestinal issues;
- Existing research has primarily focused on the association between khat chewing and elevated levels of hepatocellular enzymes (like ALT, AST), suggesting potential liver cell damage.

### What this study adds

- This study provides new evidence that chronic Khat chewing is associated with significant alterations in the liver's synthetic and excretory functions, specifically showing elevated total bilirubin and reduced total protein levels, which were not the focus of prior studies;
- It highlights that while certain liver functions are affected (bilirubin and protein), others like albumin and GPT levels remain unchanged in this population, offering a more nuanced understanding of Khat's hepatotoxic profile.
